# Effectiveness of processing laryngoscopes used in surgical patients undergoing orotracheal intubation

**DOI:** 10.1590/0034-7167-2025-0167

**Published:** 2026-01-26

**Authors:** Luiz Miguel de Paula Moura, Adélia Dayane Guimarães Fonseca, Luciane Ribeiro, Camila Silva Torres Militão, Adriely de Abreu Varoto, Fábio da Costa Carbogim, Álvaro Francisco Lopes de Sousa, André Luiz Silva Alvim

**Affiliations:** I Universidade Federal de Juiz de Fora. Juiz de Fora, Minas Gerais, Brazil; II Universidade Federal de Juiz de Fora, Hospital Universitário. Juiz de Fora, Minas Gerais, Brazil; III Hospital Sírio-Libanês, Instituto de Ensino e Pesquisa. São Paulo, São Paulo, Brazil; IV Universidade Nova de Lisboa. Lisboa, Portugal

**Keywords:** Disinfection, Equipment Reuse, Laryngoscopes, Intubation, Endotracheal, Infection Control., Desinfección, Equipos Reutilizables, Laringoscopio, Intubación Endotraqueal, Control de Infecciones.

## Abstract

**Objectives:**

to evaluate the effectiveness of processing laryngoscopes used in surgical patients undergoing orotracheal intubation.

**Methods:**

this was a descriptive, analytical, quantitative study conducted in a public hospital in Minas Gerais, Brazil, in two phases: assessment of the protein test and the persistence of simulated microorganisms on laryngoscopes. Data were analyzed using descriptive statistics and the Wilcoxon Signed Rank test.

**Results:**

a total of 48 samples were analyzed, distributed across 24 protein tests, 12 fluorescence analyses, and 12 visual inspections of the laryngoscope conditions. There was a significant reduction in protein levels after processing (z = -2.58, p < 0.010), with a moderate effect size (r = 0.74). However, all samples failed the qualitative assessment, as protein levels exceeded the acceptable limit of 1 µg/cm^2^.

**Conclusions:**

there was a reduction in simulated microorganisms. However, all protein tests failed, indicating the ineffectiveness of laryngoscope disinfection.

## RESUMEN

**Objetivos:**

evaluar la efectividad del procesamiento de laringoscopios utilizados en pacientes quirúrgicos sometidos a intubación orotraqueal.

**Métodos:**

se trata de un estudio descriptivo, analítico y de naturaleza cuantitativa, realizado en un hospital público de Minas Gerais, en dos fases: evaluación de la prueba de proteína y de la persistencia de microorganismos simulados en los laringoscopios. Los datos fueron analizados mediante estadística descriptiva y la prueba de *Wilcoxon Signed Rank*.

**Resultados:**

se analizaron 48 muestras, distribuidas en 24 pruebas de proteína, 12 análisis por fluorescencia y 12 inspecciones visuales de las condiciones de los laringoscopios. Hubo una reducción significativa en los niveles de proteína después del procesamiento (z = -2,58, p < 0,010), con un tamaño de efecto moderado (r = 0,74). Sin embargo, todas las muestras fueron reprobadas cualitativamente, ya que los niveles de proteína superaron el límite aceptable de 1 µg/cm^2^.

**Conclusiones:**

hubo una reducción de los microorganismos simulados. No obstante, todas las pruebas de proteína fueron reprobadas, lo que indica ineficacia en la desinfección de los laringoscopios.

## INTRODUCTION

The processing of medical devices (PMD) is essential for the decontamination and preparation of materials and equipment used in surgeries, care procedures, and diagnostic exams. Performed mainly by the nursing team in the Central Sterile Supply Department (CSSD), this process ensures the safe reuse or proper disposal of items in healthcare services^(1)^. In certain areas, such as the surgical center, the initial cleaning and disinfection of semicritical PMD can be performed locally to optimize workflow and minimize the risk of cross-contamination^(2)^.

In this context, PMD are classified into three categories: critical, semicritical, and noncritical. Critical devices are those that penetrate sterile tissues or organs, presenting a high risk of infection and therefore requiring sterilization. Semicritical devices come into contact with mucous membranes or non-intact skin, requiring high-level disinfection or sterilization. Noncritical devices, on the other hand, come into contact only with intact skin, requiring only low-level disinfection^(1,3)^. It is important to emphasize that proper classification is essential for choosing the appropriate disinfection or sterilization method, ensuring patient safety and the effectiveness of procedures^(4)^.

Multiple steps are involved in PMD processing, including pre-cleaning, cleaning, inspection, preparation, disinfection or sterilization, and storage. These steps apply to a wide variety of items, such as surgical instruments, endoscopes, forceps, and other materials used in various procedures. Each phase of processing plays a fundamental role in removing debris, microorganisms, and both organic and inorganic matter, contributing to the prevention of healthcare-associated infections (HAIs). Thus, healthcare professionals involved must follow strict protocols to ensure the safety and quality of the various processes, according to the guidelines established by the Brazilian National Health Surveillance Agency (Anvisa)^(1,5)^.

In the surgical center, it is common for some semicritical devices to undergo cleaning and disinfection locally before being sent for the final stage at the CSSD, and, in some cases, they may remain in the unit for subsequent use. An example is the laryngoscope, which consists of a handle containing batteries to power a light bulb and a straight or curved blade, whose processing must follow validated protocols. However, keeping these devices in the unit requires strict control measures, including process traceability, validation of the methods employed, and compliance with recommended protocols for the safe reuse of these medical devices^(6)^.

The laryngoscope is a device, generally made of stainless steel or brass and heat-resistant, essential for tracheal intubations, airway observation, and surgical procedures on the larynx. Due to its frequent use, its cleaning and disinfection can be performed in satellite units, such as the surgical center itself, ensuring greater agility in care. In some settings, this process is carried out by the nursing team, while in others, it is performed by anesthesiologists, depending on the organization and workflow of the healthcare service^(6,7)^.

During laryngoscopy, the blade of the laryngoscope is introduced into the oral cavity, being classified as a semicritical item, as it comes into contact with mucous membranes, saliva, and occasionally blood, which can contaminate not only the blade but also the handle. Both components must undergo thorough processing before being reused on another patient. This process necessarily includes initial cleaning and, at a minimum, intermediate-level disinfection, which can be performed with cleaning agents that comply with current sanitary regulations^(7)^.

The literature^(7,8)^ points to a significant gap in the understanding of healthcare professionals involved in laryngoscope disinfection, highlighting low adherence to protocols and lack of familiarity with the procedures and products used, which may increase the risk of incidents and adverse events. However, it is noted that researchers focus mainly on the team’s knowledge, without evaluating how the process actually occurs in practice, justifying the need for this study. A study by the Spanish Society of Anesthesiology, conducted with 248 anesthesiologists, revealed that 31.5% do not follow a disinfection protocol, 45% are unaware of standard operating procedures (SOPs), and 87% do not know which detergent is used. In addition, 50% of services do not properly disinfect laryngoscope handles, and only 16% store these devices in sealed containers^(8)^.

In this context, the guiding questions of this research arise: How effective is the processing of laryngoscopes used in anesthetic care in the surgical center? Does the proper use of these medical devices effectively contribute to the prevention of HAIs? The importance of this study for nursing lies in the critical analysis of the conformity of cleaning and disinfection practices for these devices in the hospital environment. By investigating the effectiveness of laryngoscope processing, the aim is to identify failures between recommended protocols and their practical application in healthcare services. This approach enables improvements in processes, with direct impacts on patient safety, the quality of anesthetic care, and infection control, reinforcing the strategic role of nursing in promoting safe, evidence-based care.

## OBJECTIVES

To evaluate the effectiveness of processing laryngoscopes used in surgical patients undergoing orotracheal intubation.

## METHODS

### Ethical aspects

This study is part of the umbrella project entitled “Analysis of the Practices of Professionals in the Central Sterile Supply Department: Processing of Medical Devices”, approved by the Research Ethics Committee. As this was a laboratory-based study, it was not necessary to obtain informed consent.

### Study design

This is a descriptive, analytical, quantitative study. The Standards for Quality Improvement Reporting Excellence (SQUIRE 2.0) instrument was adopted to guide the writing of this research, providing a framework for transparent and detailed reporting of the methods and results of improvement initiatives, thus allowing for their evaluation, replication, or adaptation to different contexts^(9)^.

### Study setting

The study was conducted in a medium-sized general hospital located in the Zona da Mata region of Minas Gerais. The institution is a referral center for patients from the Unified Health System (SUS in Portuguese), with medical-surgical specialties including general surgery, urology, gynecology, orthopedics, and oncology.

### Study protocol

To determine the type of processing applied to each laryngoscope, the devices were randomly selected, regardless of the surgical procedure in which they had been used. The first laryngoscope to be processed was randomly assigned for assessment of the cleaning and disinfection methods employed, and this distribution was maintained throughout the entire data collection period. This process was repeated weekly, always between Wednesday and Friday, which were the days when the surgical center had the highest volume of procedures. All laryngoscopes processed in the hospital during the established data collection days and within the study period comprised the sample.

The inclusion criteria consisted of laryngoscopes used in surgical procedures requiring general anesthesia with orotracheal intubation, as this type of intervention ensured that the devices had been exposed to actual biological material, reflecting typical conditions of use. New laryngoscopes that had been used for the first time were excluded, since these devices, having not undergone repeated cycles of use and processing, could present contamination and wear levels different from those of instruments in regular circulation in the service, which would compromise the standardization of the analysis.

**Figure 1:**
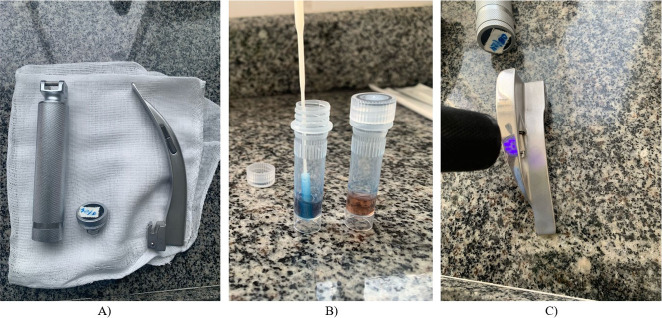
Study phases using the protein test and the fluorescence method, Juiz de Fora, Minas Gerais, Brazil, 2025

Data collection was carried out over eight weeks, beginning in January and ending in February 2025, and took place in two phases (Figure 1). In phase 1, a protein test was performed to assess the effectiveness of the cleaning methods. The test was carried out using STC-W115 brand cleaning indicators to detect protein residues and was followed by the application of Optoglow^®^ fluorescent solution, which simulated the persistence of microorganisms on previously contaminated laryngoscopes. Subsequently, the devices were subjected to the processing method routinely used in the institution, which consisted of manual cleaning with enzymatic detergent and disinfection with 70% alcohol. After processing, phase 2 consisted of a new protein test assessment using the same laryngoscope, in order to investigate the impact of the adopted cleaning and disinfection process. The fluorescent solution was checked using a fluorescent flashlight provided by the same supplier. At all times, the researchers used personal protective equipment.

*A) Random selection of laryngoscopes was performed after use in surgical procedures with anesthetic assistance; B) Phase 1 - protein test, with both qualitative and quantitative readings conducted before and after processing; C) Phase 2 - verification of the presence of fluorescent solution after processing.*

The research instrument was developed by the investigators to evaluate the effectiveness of the processing. Variables analyzed included the processing method, the use of sponges versus gauze pads, environmental conditions in the decontamination area, and the presence of protein residues and simulated microorganisms before and after cleaning and disinfection. Temperature and humidity in the decontamination area were recorded, as this was the designated location for the procedure.

The assessment took place in two phases. Before disinfection, an analysis of protein residues was performed, quantifying the residual load (µg/cm^2^) in ordinal values and subsequently classifying it as approved or not approved. In this case, values above 1 µg/cm^2^ indicated failure of the laryngoscope. After disinfection, in addition to repeating the protein test to assess the impact of processing, the physical condition of the laryngoscopes was evaluated, including the presence of visible dirt, stains, oxidation, corrosion, and changes in coloration. The persistence of the fluorescent solution was assessed using a fluorescent flashlight provided by the same manufacturer and classified as present or absent.

### Data analysis

Data were analyzed using descriptive statistics to present absolute and relative values. The normality of the variables was verified using the Shapiro-Wilk test. To compare the results of the protein test before and after laryngoscope processing (residual load values in µg/cm^2^), the Wilcoxon Signed Rank test was used, with a significance level set at 5%.

## RESULTS

A total of 48 samples (100%) were analyzed, distributed as follows: 24 (50%) protein tests conducted in both study phases, 12 (25%) fluorescence assessments, and 12 (25%) visual inspections to verify the condition of the laryngoscopes after processing.

Of the 12 laryngoscopes evaluated during the manual cleaning phase, 9 (75%) were cleaned using specific non-abrasive sponges intended for this purpose. In 3 (25%) laryngoscopes, cleaning was performed with gauze pads that had been left exposed in the decontamination area, which is designated for the processing of contaminated materials.

**Figure 2:**
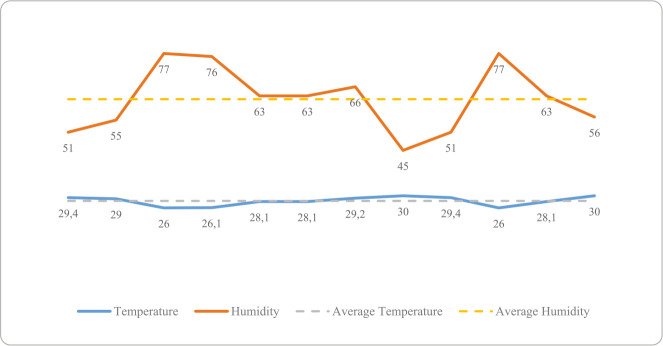
Assessment of temperature and humidity in the decontamination area of the study hospital, Juiz de Fora, Minas Gerais, Brazil, 2025

Figure 2 shows the conditions in the decontamination area with respect to temperature and humidity. Recorded temperatures ranged from 26°C to 30°C, with an average of 28°C. Humidity values fluctuated between 45% and 77%, with an average of 62%.

Descriptive analysis of the results shows that all laryngoscopes tested positive for the presence of proteins in phase 1, with values ranging from 2 to 10 µg/cm^2^. In phase 2, there was a reduction in protein levels, with concentrations ranging from 2 to 8 µg/cm^2^. Regarding the fluorescence assessment, no solution residues were identified in any of the samples, indicating the elimination of the simulated microorganisms. When analyzing the condition of the laryngoscopes after disinfection by visual inspection, it was found that 25% (n=3) of the samples showed alterations (Table 1).

**Table 1:** Description of the protein test values, fluorescence methods, and conditions of the laryngoscopes after the disinfection process, Juiz de Fora, Minas Gerais, Brazil, 2025 (n=12)

Sample	Protein Test (µg/cm^2^)		Presence of Fluorescent Solution^*^	Conditions after Disinfection^**^
	Step 1	Step 2		
Laryngoscope 1	10	02	Absent	No changes
Laryngoscope 2	08	02	Absent	No changes
Laryngoscope 3	04	02	Absent	No changes
Laryngoscope 4	08	08	Absent	With visible dirt
Laryngoscope 5	08	08	Absent	No changes
Laryngoscope 6	04	04	Absent	With visible dirt
Laryngoscope 7	04	02	Absent	No changes
Laryngoscope 8	04	02	Absent	No changes
Laryngoscope 9	10	02	Absent	No changes
Laryngoscope 10	04	02	Absent	No changes
Laryngoscope 11	04	04	Absent	With visible dirt
Laryngoscope 12	04	02	Absent	No changes

* Fluorescence methods;

** Performed by visual inspection.e

**Figure 3:**
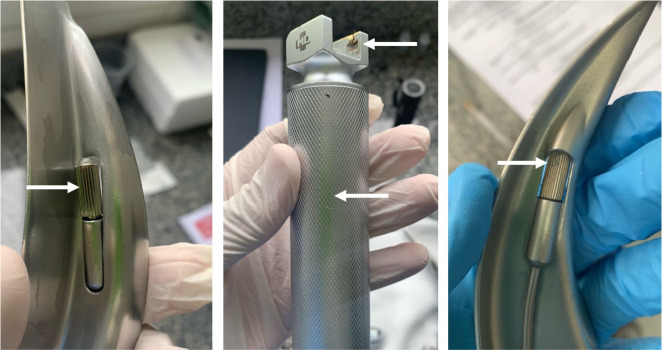
Alterations identified in laryngoscopes after the cleaning and disinfection process, Juiz de Fora, Minas Gerais, Brazil, 2025

Among the alterations identified in the laryngoscopes, the presence of visible dirt was most notable, especially on the bulbs responsible for illuminating the airway, located on the blades, as well as on the handle of the laryngoscope, specifically in the area of handling and at the blade attachment site (Figure 3).

The results were statistically significant (z = -2.58, p < 0.010), demonstrating that protein residue levels were lower after laryngoscope processing (Table 2), with a moderate effect size (r = 0.74). However, it is important to note that, qualitatively-meaning when considering only the classification as positive or negative-100% of the samples failed both in phase 1 and phase 2, since values equal to or greater than 1 µg/cm^2^ are considered noncompliant.

**Table 2:** Descriptive statistics for protein test values in phases 1 and 2, Juiz de Fora, Minas Gerais, Brazil, 2025

Evaluation	Mean	SD	Median
Phase 1	6.0	2.55	4.0
Phase 2	3.3	2.3	2.0

*SD = Standard deviation; z = -2.58, p < 0.010.*

## DISCUSSION

The analysis of the results showed that all laryngoscopes had protein residues above the acceptable limit in the first phase, as expected due to their recent use in intubations. Although there was a reduction in the second phase, no sample achieved compliance in the qualitative assessment, highlighting failures in the removal of organic matter. From a quantitative perspective, it is important to note that there was a decrease in values; however, the residues remained above 1 µg/cm^2^. The fluorescence assessment confirmed the elimination of simulated microorganisms; however, visual inspection revealed dirt in some critical areas, such as the blades and fittings.

In this study, temperature and humidity showed significant fluctuations during the data collection period. There is no consensus on the ideal conditions for these two indicators in the decontamination areas of surgical centers. ANVISA recommends that the cleaning room of the CSSD maintain a temperature between 18°C and 22°C, with humidity between 40% and 60%^(3)^, a guideline that can be applied to satellite units such as the surgical center. However, these recommendations vary and often lack solid theoretical support. In this regard, the Association for the Advancement of Medical Instrumentation suggests a maximum limit of 24°C, arguing that this temperature promotes the comfort of professionals and reduces the proliferation of microorganisms, which tend to grow more in warmer environments^(10)^. Considering both guidelines, it should be noted that a variation between 26°C and 30°C indicates the need for an action plan to reduce the temperature.

Despite this, uncertainties remain about the true influence of temperature and humidity on the safety of laryngoscopes. Observations in the study setting revealed challenges in maintaining these parameters, especially in controlling relative air humidity, which can compromise the quality of PMD processing and the safety of the professionals involved. In this latter case in particular, a study highlights that maintaining relative air humidity between 40% and 60% improves airway health, reduces the viability of infectious viruses, and decreases the risk of respiratory infections and acute symptoms^(11)^.

This study showed that, although there was a reduction in protein levels in phase 2, all samples failed qualitatively, indicating the presence of residues in all laryngoscopes. Researchers have developed an innovative method to evaluate the effectiveness of cleaning and have reinforced the importance of precise techniques for detecting protein residues. Various assessment methods and their respective accuracies in detecting protein residues were analyzed, with all tested protocols demonstrating similar sensitivity and precision. One of the methods used, which applied residual protein to materials to investigate contamination on their surfaces, revealed that all twelve PMD had protein residues, indicating dirt and corroborating the findings of this study^(12)^.

In this context, some modifications were observed in the laryngoscope decontamination process that did not follow the standards required by the institution’s SOP. These modifications occurred mainly due to a lack of supplies and materials necessary for proper processing. Laryngoscope decontamination was performed manually, using sponges and, on some occasions, gauze pads that were left exposed in the decontamination area for cleaning medical devices. This practice may result in variations in efficacy due to the subjective nature of the manual process, which can lead to the detection of protein residues after cleaning and disinfection, thereby compromising the effectiveness of the cleaning and disinfection process.

The use of gauze pads exposed in the decontamination area for the manual cleaning of laryngoscopes, instead of the recommended non-abrasive sponges, constitutes a practice inconsistent with current Brazilian regulations. National legislation establishes that this step must follow strict protocols, including the use of appropriate materials and performing cleaning under controlled conditions, in order to ensure the efficient removal of debris and minimize the risk of cross-contamination. The use of reused gauze pads stored in a potentially contaminated environment compromises the effectiveness of the cleaning step, resulting in residual protein, as evidenced in the present study. Such a procedure undermines the safety of the process and does not comply with the regulatory principles that guide quality assurance and safety of medical devices in Brazil^(3)^.

A survey on laryngoscope decontamination techniques, conducted through an online questionnaire answered by 73 anesthesiologists from 37 major tertiary care centers in India, revealed a lack of uniformity in the methods adopted, both among different institutions and within the same institution^(13)^. Therefore, it can be inferred that clear protocols and adequate training are essential, since, as observed in various healthcare services-including the present study-there is neither standardization nor sufficient training for professionals who deal with laryngoscope processing on a daily basis.

Another step in the study protocol involved applying the fluorescent solution test to all laryngoscopes included in the investigation. Although manual cleaning did not follow the recommended standards, all devices showed negative results, with no detection of the solution after the cleaning and disinfection process. Researchers suggest that the fluorescent solution is an effective tool for identifying failures in the cleaning process, indicating that manual cleaning combined with 70% alcohol is more effective than automated cleaning^(9)^. In addition, the use of simulators for biological material contamination has proven effective in evaluating the decontamination of medical devices, as demonstrated in another study^(14-16)^. It is recommended that the fluorescence method be considered by healthcare services as an effective alternative for use in team training, aiming to raise awareness about the importance of quality in the decontamination process.

Although manual cleaning followed by scrubbing with 70% alcohol is considered an effective disinfection process in most cases, the present study identified visible dirt on some laryngoscopes that underwent this procedure. This limitation highlights the need for other validated disinfection methods or the use of hydrogen peroxide, especially for semicritical equipment. One study proposed a quantitative method to assess patient risk associated with the use of medical devices, emphasizing the importance of appropriate cleaning protocols to minimize infection risks. Among the findings, it is noteworthy that the specific characteristics of the processed material directly influence the effectiveness of disinfection, highlighting the need for specific approaches for each item^(15)^.

Considering that medical devices must undergo rigorous cleaning and disinfection processes to eliminate most contamination, it is notable that the laryngoscope carries a significant microbial and organic load. For this reason, it is essential that these devices, along their entire length-from blade to handle-undergo proper processing. An integrative review study discusses that the handle of the laryngoscope is often neglected in this process, which is a mistake, since the handle can be a point of cross-contamination due to hand contact during use in the surgical center, compromising patient safety^(6)^.

The analysis of the effectiveness of laryngoscopes after use made it possible to verify the efficacy of protein residue removal and the persistence of contamination using the method adopted in the healthcare service, providing support for evaluating the safety of the protocols used. These findings demonstrate that, although there was a reduction in the levels measured and elimination of simulated microorganisms, cleaning and disinfection were not fully effective in removing organic residues, reinforcing the need for review and improvement of the institutional protocol.

### Study limitations

This study has relevant methodological limitations. The evaluation was conducted on only 12 laryngoscopes, a small sample size that compromises external validity and limits the generalizability of the results to other populations or contexts. Future research should increase the sample size to strengthen the robustness of the findings. Additionally, measurement bias may have occurred due to heterogeneity in the application of the cleaning protocol, resulting from the lack of standardized training among professionals. The lack of control over operational confounding factors, such as the temporary unavailability of essential supplies, compromised process uniformity and impacted the results.

### Contributions to the Field

This study offers significant contributions to nursing clinical practice by providing an in-depth analysis of the processing of laryngoscopes used during anesthetic care. The findings highlighted deficiencies in manual disinfection procedures, such as the presence of protein residues above acceptable limits, signaling the need for more standardized and effective methods. These results support the revision and strengthening of institutional protocols for medical device reprocessing, with direct implications for patient safety and the protection of healthcare professionals during tracheal intubation. Additionally, they underscore the importance of nursing team involvement in adopting best practices and promoting continuous training, reinforcing their essential role in infection prevention and in ensuring quality care in surgical settings.

## CONCLUSIONS

This study evaluated the effectiveness of processing laryngoscopes used in surgical patients undergoing orotracheal intubation. The fluorescence assessment indicated the elimination of simulated microorganisms, suggesting the effectiveness of the disinfection process from a microbiological perspective. Although a reduction in protein levels was observed after processing, the values remained above the acceptable limit. All samples were qualitatively evaluated and failed in the second phase of the study, as levels remained above 1 µg/cm^2^. Furthermore, the presence of visible dirt in some samples after processing indicates that cleaning was not completely effective in removing organic residues.

## Data Availability

The research data are available within the article.

## References

[1] Agência Nacional de Vigilância Sanitária (Anvisa) (2020). Tema 15.3 - Boas práticas para o processamento de produtos para saúde.

[2] Link T. (2021). Guidelines in practice: instrument cleaning. AORN J.

[3] Agência Nacional de Vigilância Sanitária (Anvisa) (2012). Dispõe sobre as boas práticas para o processamento de produtos para a saúde.

[4] Rowan NJ, Kremer T, McDonnell G. (2023). A review of Spaulding's classification system for effective cleaning, disinfection and sterilization of reusable medical devices: viewed through a modern-day lens that will inform and enable future sustainability. Sci Total Environ.

[5] Zheng S, Jiang D, Liu P, Zhang H. (2023). Management Quality of surgical instrument and influence of cleaning and sterilization on the surgical outcomes of the patient: a review. Altern Ther Health Med.

[6] Bruna QMC, Oliveira AC, Mendes KDS, Mazzo A, Silva DMP, Godoy S. (2016). Processamento de cabos de laringoscópio: revisão integrativa. Rev SOBECC.

[7] Lasaponari E, Almeida M, Pires N, Oliveira G. (2018). In: 11º Simpósio Internacional de Esterilização e Controle de Infecção Relacionada à Saúde.

[8] Gómez-Ríos MÁ, Sastre JA, López T, Gaszyński T. (2023). Disinfection of reusable laryngoscopes: a survey about the clinical practice in Spain. Healthcare (Basel).

[9] Mineli TA, Andrade D, Godoy S, Mendes IAC, Tognoli SH, Marchi-Alves LM. (2021). Reuse of hospital bedpans. Rev Bras Enferm.

[10] Association for Advancement of Medical Instrumentation (AAMI); American National Standards (2006). Comprehensive guide to steam sterilization and sterility assurance in health care facilities.

[11] Wolkoff P. (2024). Indoor air humidity revisited: impact on acute symptoms, work productivity, and risk of influenza and COVID-19 infection. Int J Hyg Environ Health.

[12] Ouirungroj T, Apichai S, Pattananandecha T, Grudpan K, Saenjum C. (2024). Smart-detection approach for protein residues to evaluate the cleaning efficacy of reusable medical devices. J Hosp Infect.

[13] Chawla R, Gupta A, Gupta A, Kumar M. (2016). Laryngoscope decontamination techniques: a survey. J Anaesthesiol Clin Pharmacol.

[14] Carvalho AA, Girondi JBR, Sebold LF, Amante LN, Alvarez AG, Waterkemper R. (2021). Melhores práticas de reprocessamento de produtos para saúde. Rev SOBECC.

[15] Kremer T, Rowan NJ, McDonnell G. (2025). A new quantitative method for determining patient risk for reusable medical device categorization based on using and interpreting Kremer's cleaning classification system. J Hosp Infect.

[16] Alvim ALS. (2023). Assessment of soiling on highly touched clinical surfaces in intensive care units. Florence Nightingale J Nurs.

