# Supplementary material for: Effectiveness of processing laryngoscopes used in surgical patients undergoing orotracheal intubation
Source: Rev Bras Enferm. 2026 Jan 26;78(6):e20250167. doi: 10.1590/0034-7167-2025-0167 (PMC12854265; doi:10.1590/0034-7167-2025-0167)
Supplement: Translation

# Translation: Efetividade do processamento de laringoscópios utilizados em pacientes cirúrgicos submetidos a intubação orotraqueal

**DOI:** 10.1590/0034-7167-2025-0167pt

**Authors:** Luiz Miguel de Paula Moura, Adélia Dayane Guimarães Fonseca, Luciane Ribeiro, Camila Silva Torres Militão, Adriely de Abreu Varoto, Fábio da Costa Carbogim, Álvaro Francisco Lopes de Sousa, André Luiz Silva Alvim

**Affiliations:** I Universidade Federal de Juiz de Fora. Juiz de Fora, Minas Gerais, Brasil; II Universidade Federal de Juiz de Fora, Hospital Universitário. Juiz de Fora, Minas Gerais, Brasil; III Hospital Sírio-Libanês, Instituto de Ensino e Pesquisa. São Paulo, São Paulo, Brasil; IV Universidade Nova de Lisboa. Lisboa, Portugal

**Keywords:** Desinfecção, Equipamentos Reutilizáveis, Laringoscópio, Intubação Endotraqueal, Controle de Infecções.

## Abstract

**Objetivos:**

avaliar a efetividade do processamento de laringoscópios utilizados em pacientes cirúrgicos submetidos a intubação orotraqueal.

**Métodos:**

trata-se de um estudo descritivo, analítico, de natureza quantitativa, realizado em um hospital público de Minas Gerais, em duas fases: avaliação do teste de proteína e da permanência de microrganismos simulados em laringoscópios. Os dados foram analisados por estatística descritiva e pelo teste de *Wilcoxon Signed Rank*.

**Resultados:**

foram 48 amostras, distribuídas em 24 testes de proteína, 12 análises por fluorescência e 12 inspeções visuais das condições dos laringoscópios. Houve redução significativa nos níveis de proteína após o processamento (*z* = -2,58, *p* < 0,010), com tamanho de efeito moderado (*r* = 0,74). Contudo, todas as amostras foram reprovadas qualitativamente, pois os níveis de proteína foram superiores ao limite aceitável de 1 µg/cm^2^.

**Conclusões:**

houve redução dos microrganismos simulados. Contudo, todos os testes de proteína foram reprovados, indicando ineficácia da desinfecção dos laringoscópios.

## INTRODUÇÃO

O processamento de Produtos para Saúde (PPS) é essencial para a descontaminação e preparação de materiais e equipamentos utilizados em cirurgias, procedimentos assistenciais e exames diagnósticos. Realizado principalmente pela equipe de enfermagem no Centro de Materiais e Esterilização (CME), garante a reutilização segura ou o descarte adequado nos serviços de saúde^(1)^. Em determinados setores, como o centro cirúrgico, a limpeza inicial e a desinfecção de PPS semicríticos podem ser realizadas localmente para otimizar o fluxo e minimizar o risco de contaminação cruzada^(2)^.

Nesse contexto, os PPS são classificados em três categorias: críticos, semicríticos e não críticos. Os produtos críticos são aqueles que penetram tecidos ou órgãos estéreis, apresentando alto risco de infecção e, por isso, exigem a esterilização. Já os semicríticos entram em contato com mucosas ou pele não íntegra, demandando desinfecção de alto nível ou esterilização. Por fim, os não críticos são aqueles que tocam apenas a pele íntegra, requerendo apenas desinfecção de baixo nível^(1,3)^. Vale ressaltar que a correta classificação é fundamental para a escolha adequada do método de desinfecção ou esterilização, garantindo a segurança do paciente e a eficácia dos procedimentos^(4)^.

São múltiplas etapas envolvidas no processamento de PPS, incluindo a pré-limpeza, a limpeza, a inspeção, o preparo, a desinfecção ou esterilização e o armazenamento. Elas são aplicáveis a uma ampla variedade de itens, como instrumentais cirúrgicos, endoscópios, pinças e outros materiais utilizados nos diversos procedimentos. Cada fase do processamento desempenha um papel fundamental na remoção de sujidades, microrganismos, bem como de matéria orgânica e inorgânica, contribuindo para a prevenção das infecções relacionadas à assistência à saúde (IRAS). Dessa forma, os profissionais de saúde envolvidos devem seguir protocolos rigorosos, garantindo a segurança e a qualidade dos diversos processos, seguindo as diretrizes estabelecidas pela Agência Nacional de Vigilância Sanitária^(1,5)^.

No centro cirúrgico, é comum que alguns dispositivos semicríticos passem por limpeza e desinfecção localmente antes do envio para a etapa final no CME, podendo, em alguns casos, permanecer no próprio setor para uso subsequente. Um exemplo é o laringoscópio, composto por um cabo, que contém pilhas para alimentar uma lâmpada, e uma lâmina reta ou curva, cujo processamento deve seguir protocolos validados. Contudo, a permanência desses dispositivos no setor exige medidas rigorosas de controle, incluindo a rastreabilidade do processo, a validação dos métodos empregados e a conformidade com os protocolos recomendados para a reutilização segura desses produtos para saúde^(6)^.

O laringoscópio é um equipamento, geralmente feito de aço inoxidável ou latão e termorresistente, essencial para intubações traqueais, observação das vias respiratórias e procedimentos cirúrgicos na laringe. Devido à sua utilização frequente, sua limpeza e desinfecção podem ser realizadas em unidades satélites, como o próprio centro cirúrgico, garantindo maior agilidade no atendimento. Em alguns locais, esse processo é conduzido pela equipe de enfermagem, enquanto, em outros, é realizado pelos anestesistas, dependendo da organização e do fluxo de trabalho do serviço de saúde^(6,7)^.

Durante a laringoscopia, a lâmina do laringoscópio é introduzida na cavidade oral, sendo classificada como material semicrítico, uma vez que entra em contato com mucosas, saliva e, ocasionalmente, sangue, o que pode contaminar não apenas a lâmina, mas também o cabo. Ambos devem passar por um rigoroso processamento antes de serem reutilizados em outro paciente. Esse processo inclui, obrigatoriamente, a limpeza inicial e, no mínimo, a desinfecção de nível intermediário, que pode ser realizada com produtos saneantes que estejam em conformidade com as normativas sanitárias vigentes^(7)^.

A literatura^(7,8)^ aponta uma lacuna significativa na compreensão dos profissionais de saúde envolvidos na desinfecção de laringoscópios, evidenciando baixa adesão aos protocolos e falta de familiaridade com os procedimentos e produtos utilizados, o que pode aumentar o risco de incidentes e eventos adversos. No entanto, observa-se que os pesquisadores focam principalmente no conhecimento da equipe, sem avaliar como o processo ocorre na prática, justificando a necessidade deste estudo. Um estudo da Sociedade Espanhola de Anestesiologia, realizado com 248 anestesiologistas, revelou que 31,5% não seguem um protocolo de desinfecção, 45% desconhecem os procedimentos operacionais padrão (POP) e 87% não sabem qual detergente é utilizado. Além disso, 50% dos serviços não realizam a desinfecção adequada dos cabos de laringoscópios, e apenas 16% armazenam esses dispositivos em recipientes selados^(8)^.

Nesse contexto, surgem as questões norteadoras desta pesquisa: Qual é a efetividade do processamento de laringoscópios utilizados na assistência anestésica em centro cirúrgico? A utilização adequada desses produtos para a saúde contribui efetivamente para a prevenção das IRAS? A importância deste estudo para a Enfermagem reside na análise crítica da conformidade das práticas de limpeza e desinfecção desses dispositivos no ambiente hospitalar. Ao investigar a efetividade do processamento dos laringoscópios, busca-se identificar falhas entre os protocolos recomendados e sua aplicação prática nos serviços de saúde. Essa abordagem permite propor melhorias nos processos, com impactos diretos na segurança do paciente, na qualidade da assistência anestésica e no controle de infecções, reforçando o papel estratégico da Enfermagem na promoção de cuidados seguros e baseados em evidências.

## OBJETIVOS

Avaliar a efetividade do processamento de laringoscópios utilizados em pacientes cirúrgicos submetidos a intubação orotraqueal.

## MÉTODOS

### Aspectos éticos

Este estudo integra o projeto guarda-chuva intitulado “Análise das Práticas de Profissionais do Centro de Materiais e Esterilização: Processamento de Produtos para Saúde”, aprovado pelo Comitê de Ética em Pesquisa (CEP). Como se trata de um estudo laboratorial, não foi necessário obter Consentimento Livre e Esclarecido.

### Delineamento

Trata-se de um estudo descritivo, analítico, de natureza quantitativa. Para nortear a escrita desta pesquisa, foi adotado o instrumento *Standards for Quality Improvement Reporting Excellence* (SQUIRE 2.0), que fornece uma estrutura para relatar de forma transparente e detalhada os métodos e resultados de iniciativas de melhoria, permitindo sua avaliação, replicação ou adaptação a diferentes contextos^(9)^.

### Local de estudo

O estudo foi realizado em um hospital geral, de médio porte, localizado na região da Zona da Mata Mineira. A instituição é uma referência no atendimento a pacientes do Sistema Único de Saúde (SUS), com especialidades médico-cirúrgicas, incluindo cirurgia geral, urologia, ginecologia, ortopedia e oncologia.

### Protocolo de estudo

Para determinar o tipo de processamento aplicado a cada laringoscópio, foi realizada uma seleção aleatória dos dispositivos, independentemente do procedimento cirúrgico em que foram utilizados. O primeiro laringoscópio a ser processado foi designado aleatoriamente para avaliação dos métodos de limpeza e desinfecção empregados, e essa distribuição foi mantida ao longo de todo o período de coleta de dados. Esse processo foi repetido semanalmente, sempre entre quarta e sexta-feira, dias em que o centro cirúrgico apresentava maior fluxo de procedimentos. Todos os laringoscópios processados no hospital, durante os dias estabelecidos para a coleta de dados e dentro do período do estudo, compuseram a amostra.

Os critérios de inclusão compreenderam os laringoscópios utilizados em procedimentos cirúrgicos que exigiram anestesia geral com intubação orotraqueal, uma vez que esse tipo de intervenção garante que os dispositivos tenham sido expostos a material biológico real, refletindo as condições habituais de uso. Foram excluídos os laringoscópios novos, que haviam sido utilizados pela primeira vez, uma vez que esses dispositivos, por não terem passado por ciclos repetidos de uso e processamento, poderiam apresentar níveis de contaminação e desgaste distintos dos instrumentos em circulação no serviço, o que comprometeria a padronização da análise.

A coleta de dados foi realizada ao longo de oito semanas, iniciando em janeiro e finalizando em fevereiro de 2025, e ocorreu em duas fases (Figura 1). Na etapa 1, foi avaliado o teste de proteína para justificar a eficácia dos métodos de limpeza. O teste foi realizado utilizando indicadores de limpeza da marca STC-W115 para detecção de resíduos de proteína e, em seguida, foi aplicada a solução fluorescente Optoglow^®^, que simulava a permanência de microrganismos nos laringoscópios previamente contaminados. Posteriormente, os dispositivos foram submetidos ao método de processamento rotineiramente utilizado na instituição, que consiste na limpeza manual com detergente enzimático e desinfecção com álcool 70%. Após o processamento, a etapa 2 consistiu em uma nova avaliação do teste de proteína, considerando o mesmo laringoscópio, visando investigar o impacto do processo de limpeza e desinfecção adotado. A solução fluorescente foi verificada por meio de uma lanterna fluorescente fornecida pelo mesmo fornecedor do produto. Em todos os momentos, os pesquisadores utilizaram os equipamentos de proteção individual.

**Figura 1:** Etapas do estudo utilizando o teste de proteína e o método de fluorescência, Juiz de Fora, Minas Gerais, Brasil, 2025

*A) Foi realizada a seleção aleatória dos laringoscópios após o uso em procedimentos cirúrgicos com assistência anestésica; B) etapa 1 - realização do teste de proteína, leitura qualitativa e quantitativa, antes e após o processamento; C) etapa 2 - verificação da presença de solução fluorescente após o processamento.*

O instrumento de pesquisa foi elaborado pelos pesquisadores para avaliar a efetividade do processamento. Foram analisadas variáveis como o método de processamento, o uso de buchas versus compressas, as condições ambientais no expurgo e a presença de resíduos proteicos e microrganismos simulados antes e após a limpeza e desinfecção. A temperatura e a umidade do expurgo foram registradas, uma vez que esse era o local destinado à realização do processo.

A avaliação ocorreu em duas etapas. Antes da desinfecção, foi realizada a análise de resíduos proteicos, quantificando a carga residual (µg/cm^2^) em valores ordinais e, posteriormente, classificando-a como aprovada ou reprovada. Neste caso, valores acima de 1 µg/cm^2^ indicavam a reprovação do laringoscópio. Após a desinfecção, além da repetição do teste de proteína para avaliar o impacto do processamento, foi realizada a verificação das condições físicas dos laringoscópios, incluindo a presença de sujidade visível, manchas, oxidação, corrosão e alterações na coloração. A permanência da solução fluorescente foi avaliada por meio de uma lanterna fluorescente fornecida pelo mesmo fabricante do produto, sendo classificada como presente ou ausente.

### Análise de dados

Os dados foram analisados por estatística descritiva para apresentação de valores absolutos e relativos. A normalidade das variáveis foi verificada pelo teste de Shapiro-Wilk. Para a comparação dos resultados do teste de proteína antes e após o processamento dos laringoscópios (valores da carga residual em µg/cm^2^), foi aplicado o teste de *Wilcoxon Signed Rank*, considerando um nível de significância de 5%.

## RESULTADOS

Foram analisadas 48 amostras (100%), distribuídas da seguinte forma: 24 (50%) testes de proteína nas duas etapas de estudo avaliadas, 12 (25%) avaliações por fluorescência e 12 (25%) inspeções visuais para verificar as condições de uso dos laringoscópios após o processamento.

Dos 12 laringoscópios avaliados durante a etapa de limpeza manual, 9 (75%) foram higienizados utilizando buchas não abrasivas específicas para esse fim. Já em 3 (25%) laringoscópios, a limpeza foi realizada com compressas, que estavam expostas no ambiente do expurgo, local destinado ao processamento de materiais contaminados.

A Figura 2 mostra as condições do expurgo em relação à temperatura e umidade. As temperaturas registradas variaram entre 26°C e 30°C, com uma média de 28°C. Em relação à umidade, os valores oscilaram entre 45% e 77%, obtendo uma média de 62%.

**Figura 2:** Avaliação da temperatura e umidade no expurgo do hospital de estudo, Juiz de Fora, Minas Gerais, Brasil, 2025

A análise descritiva dos resultados evidencia que todos os laringoscópios apresentaram resultado positivo para a presença de proteínas na etapa 1, com valores variando entre 2 e 10 µg/cm^2^. Na etapa 2, houve uma redução nos níveis de proteína, com concentrações variando entre 2 e 8 µg/cm^2^. Em relação à avaliação por fluorescência, não foram identificados resíduos da solução em nenhuma das amostras, o que indica a eliminação dos microrganismos simulados. Ao analisar as condições dos laringoscópios após a desinfecção, por meio da inspeção visual, verificou-se que 25% (n=3) das amostras apresentaram alterações (Tabela 1).

**Tabela 1:** Descrição dos valores do teste de proteína, métodos de fluorescência e condições dos laringoscópios após o processo de desinfecção, Juiz de Fora, Minas Gerais, Brasil, 2025 (n=12)

Amostra	Teste de proteína (µg/cm^2^)		Presença de solução fluorescente^*^	Condições após desinfecção^**^
	Etapa 1	Etapa 2		
Laringoscópio 1	10	02	Ausente	Sem alterações
Laringoscópio 2	08	02	Ausente	Sem alterações
Laringoscópio 3	04	02	Ausente	Sem alterações
Laringoscópio 4	08	08	Ausente	Com sujidade visível
Laringoscópio 5	08	08	Ausente	Sem alterações
Laringoscópio 6	04	04	Ausente	Com sujidade visível
Laringoscópio 7	04	02	Ausente	Sem alterações
Laringoscópio 8	04	02	Ausente	Sem alterações
Laringoscópio 9	10	02	Ausente	Sem alterações
Laringoscópio 10	04	02	Ausente	Sem alterações
Laringoscópio 11	04	04	Ausente	Com sujidade visível
Laringoscópio 12	04	02	Ausente	Sem alterações

* Métodos de fluorescência;

** Realizada pela inspeção visual.

Dentre as alterações identificadas nos laringoscópios, destaca-se a presença de sujidade visível, especialmente nas lâmpadas responsáveis pela iluminação da via aérea, localizadas nas lâminas, bem como no cabo do laringoscópio, especificamente na área de manuseio e no encaixe da lâmina (Figura 3).

**Figura 3:** Alterações identificadas nos laringoscópios após o processo de limpeza e desinfecção, Juiz de Fora, Minas Gerais, Brasil, 2025

Os resultados foram estatisticamente significativos (*z* = -2,58, *p* < 0,010), demonstrando que os níveis de resíduos de proteína foram menores após o processamento dos laringoscópios (Tabela 2), com tamanho de efeito moderado (*r* = 0,74). No entanto, é relevante destacar que, de forma qualitativa, ou seja, considerando apenas a classificação como positivo ou negativo, 100% das amostras foram reprovadas tanto na etapa 1 quanto na etapa 2, uma vez que valores iguais ou superiores a 1 µg/cm^2^ são considerados não conformes.

**Tabela 2:** Estatísticas descritivas para os valores do teste de proteína nas etapas 1 e 2, Juiz de Fora, Minas Gerais, Brasil, 2025

Avaliação	Média	DP	Mediana
Etapa 1	6,0	2,55	4,0
Etapa 2	3,3	2,3	2,0

*DP = Desvio-padrão; z = -2,58, p < 0,010.*

## DISCUSSÃO

A análise dos resultados mostrou que todos os laringoscópios apresentaram resíduos de proteína acima do limite aceitável na primeira etapa, como esperado devido ao uso recente em intubações. Embora tenha ocorrido redução na segunda etapa, nenhuma amostra atingiu conformidade pela avaliação qualitativa, evidenciando falhas na remoção de matéria orgânica. Do ponto de vista quantitativo, é importante mencionar que houve redução dos valores; no entanto, os resíduos permaneceram acima de 1 µg/cm^2^. A avaliação por fluorescência confirmou a eliminação dos microrganismos simulados; porém, a inspeção visual revelou sujidade em algumas áreas críticas, como lâminas e encaixes.

Neste estudo, a temperatura e a umidade apresentaram oscilações importantes durante o período de coleta de dados. Não há consenso sobre as condições ideais desses dois indicadores no expurgo dos centros cirúrgicos. A Agência Nacional de Vigilância Sanitária (Anvisa) recomenda que a sala de limpeza do CME mantenha a temperatura entre 18°C e 22°C, com umidade entre 40% e 60%^(3)^, diretriz que pode ser aplicada a unidades satélites, como o centro cirúrgico. Contudo, essas recomendações variam e, muitas vezes, carecem de embasamento teórico sólido. Nesse sentido, a *Association for the Advancement of Medical Instrumentation* sugere um limite máximo de 24°C, argumentando que essa temperatura favorece o conforto dos profissionais e reduz a proliferação de microrganismos, que tendem a crescer mais em ambientes quentes^(10)^. Considerando ambas as diretrizes, destaca-se que uma variação entre 26°C e 30°C indica a necessidade de um plano de ação para redução da temperatura.

Apesar disso, ainda há incertezas sobre a real influência da temperatura e da umidade na segurança dos laringoscópios. As observações no setor de estudo evidenciaram desafios na manutenção desses parâmetros, especialmente no controle da umidade relativa do ar, o que pode comprometer a qualidade do processamento dos PPS e a segurança dos profissionais envolvidos. Nesse último caso, em especial, um estudo destaca que manter a umidade relativa do ar entre 40% e 60% melhora a saúde das vias aéreas, reduz a viabilidade de vírus infecciosos e diminui o risco de infecções respiratórias e sintomas agudos^(11)^.

Esta pesquisa mostrou que, embora tenha ocorrido uma redução nos níveis de proteína na etapa 2, todas as amostras foram reprovadas qualitativamente, evidenciando a presença de resíduos em todos os laringoscópios. Pesquisadores desenvolveram um método inovador para avaliar a eficácia da limpeza e reforçaram a importância de técnicas precisas na detecção de resíduos proteicos. Foram analisados diversos métodos de avaliação e suas respectivas precisões na detecção de resíduos de proteínas, constatando-se que todos os protocolos testados apresentaram sensibilidade e precisão semelhantes. Um dos métodos utilizados, que aplicou proteína residual nos materiais para investigar a contaminação em suas superfícies, revelou que todos os doze PPS apresentavam resíduos de proteínas, o que indicou sujidade e corroborou os achados deste estudo^(12)^.

Nesse sentido, foram observadas algumas modificações no processo de descontaminação dos laringoscópios, que não seguiram o padrão exigido pelo POP da instituição. Essas modificações ocorreram, principalmente, devido à falta de insumos e materiais necessários para um processamento adequado. A descontaminação dos laringoscópios foi realizada manualmente, utilizando buchas e, em algumas ocasiões, compressas que ficavam expostas no expurgo para a limpeza de PPS. Essa prática pode apresentar variações na eficácia devido à subjetividade do processo manual, o que pode favorecer a detecção de resíduos de proteína após a limpeza e desinfecção, comprometendo a eficácia do processo de limpeza e desinfecção.

A utilização de compressas expostas no ambiente do expurgo para a limpeza manual dos laringoscópios, em substituição às buchas não abrasivas recomendadas, configura uma prática inconsistente com as normativas brasileiras vigentes. A legislação nacional estabelece que essa etapa deve seguir protocolos rigorosos, que incluem a utilização de materiais adequados e a realização da limpeza em condições controladas, a fim de garantir a remoção eficiente de sujidades e minimizar os riscos de contaminação cruzada. A adoção de compressas reutilizadas e armazenadas em ambiente potencialmente contaminado compromete a eficácia da etapa de limpeza, resultando em resíduos proteicos remanescentes, como evidenciado no presente estudo. Tal procedimento fragiliza a segurança do processo e está em desacordo com os princípios regulatórios que norteiam a garantia da qualidade e segurança dos produtos para saúde no Brasil^(3)^.

Um levantamento sobre as técnicas de descontaminação de laringoscópios, realizado por meio de uma pesquisa on-line respondida por 73 anestesiologistas de 37 principais centros de atendimento terciário da Índia, revelou uma ausência de uniformidade nos métodos adotados, tanto entre diferentes instituições quanto dentro da mesma instituição^(13)^. Infere-se, portanto, a importância de protocolos claros e treinamento adequado, já que, como observado em vários serviços de saúde, incluindo o do presente estudo, não há padronização nem capacitação suficiente para os profissionais que lidam diariamente com o processamento de laringoscópios.

Outra etapa do protocolo de estudo refere-se à aplicação do teste de solução fluorescente em todos os laringoscópios incluídos na investigação. Embora a limpeza manual não tenha seguido os padrões recomendados, todos os dispositivos apresentaram resultado negativo, sem a detecção da solução após o processo de limpeza e desinfecção. Pesquisadores sugerem que a solução fluorescente é uma ferramenta eficaz para identificar falhas no processo de limpeza, indicando que a limpeza manual, associada ao uso de álcool 70%, é mais eficaz do que a automatizada^(9)^. Somado a isso, a utilização de simuladores de contaminação por material biológico tem se mostrado eficaz na avaliação da descontaminação de PPS, conforme evidenciado em outro estudo^(14-16)^. Recomenda-se que o método de fluorescência seja considerado pelos serviços de saúde como uma alternativa eficaz para ser utilizada em treinamentos de equipe, visando conscientizar sobre a importância da qualidade do processo de descontaminação.

Apesar de a limpeza manual seguida de fricção com álcool 70% ser considerada um processo de desinfecção eficaz na maioria dos casos, o presente estudo identificou sujidades visíveis em alguns laringoscópios que passaram por esse procedimento. Essa limitação evidencia a necessidade de outros métodos validados de desinfecção ou do uso de peróxido de hidrogênio, especialmente para equipamentos semicríticos. Um estudo propôs um método quantitativo para avaliar o risco ao paciente associado ao uso de PPS, destacando a importância de protocolos de limpeza adequados para minimizar os riscos de infecção. Entre os achados, ressalta-se que as particularidades do material processado influenciam diretamente a eficácia da desinfecção, evidenciando a necessidade de abordagens específicas para cada item^(15)^.

Considerando que os PPS devem passar por processos rigorosos de limpeza e desinfecção para eliminar grande parte da contaminação, destaca-se que o laringoscópio apresenta uma carga microbiana e orgânica significativa. Por esse motivo, é fundamental que esses dispositivos, em toda a sua extensão, desde a lâmina até o cabo, passem por um processamento adequado. Uma pesquisa de revisão integrativa discute que, muitas vezes, o cabo do laringoscópio é negligenciado nesse processo, o que representa um erro, pois o cabo pode ser um ponto de contaminação cruzada devido ao contato das mãos do manipulador durante o uso no centro cirúrgico, comprometendo a segurança do paciente^(6)^.

A análise da efetividade dos laringoscópios após o uso permitiu verificar a eficácia da remoção dos resíduos de proteína e a persistência de contaminação por meio do método adotado no serviço de saúde, fornecendo subsídios para avaliar a segurança dos protocolos adotados. Esses achados demonstram que, embora tenha ocorrido uma redução nos níveis do teste avaliado e eliminação dos microrganismos simulados, a limpeza e desinfecção não foram totalmente eficazes na remoção de resíduos orgânicos, reforçando a necessidade de revisão e aprimoramento do protocolo institucional.

### Limitações do estudo

Este estudo apresenta limitações metodológicas relevantes. A avaliação foi realizada em apenas 12 laringoscópios, número reduzido que compromete a validade externa e restringe a extrapolação dos resultados para outras populações ou contextos. Futuras pesquisas devem ampliar a amostra para fortalecer a robustez dos achados. Adicionalmente, viés de mensuração pode ter ocorrido devido à heterogeneidade na aplicação do protocolo de limpeza, decorrente da ausência de treinamento padronizado entre os profissionais. A falta de controle sobre fatores de confusão operacionais, como a indisponibilidade temporária de insumos essenciais, comprometeu a uniformidade do processo e impactou os resultados.

### Contribuições para a Área

Este estudo oferece contribuições significativas para a prática clínica de Enfermagem ao apresentar uma análise aprofundada do processamento de laringoscópios utilizados durante a assistência anestésica. Os achados evidenciaram deficiências nos procedimentos manuais de desinfecção, como a presença de resíduos proteicos acima dos limites aceitáveis, sinalizando a necessidade de métodos padronizados e mais eficazes. Esses resultados subsidiam a revisão e o fortalecimento dos protocolos institucionais de reprocessamento de PPS, com implicações diretas na segurança do paciente e na proteção dos profissionais de saúde durante a intubação traqueal. Além disso, ressaltam a importância do envolvimento da equipe de Enfermagem na adoção de boas práticas e na promoção de capacitações contínuas, reforçando seu papel essencial na prevenção de infecções e na garantia da qualidade assistencial em ambientes cirúrgicos.

## CONCLUSÕES

Este estudo avaliou a efetividade do processamento de laringoscópios utilizados em pacientes cirúrgicos submetidos à intubação orotraqueal. A avaliação por fluorescência indicou a eliminação dos microrganismos simulados, sugerindo a eficácia do processo de desinfecção do ponto de vista microbiológico. Embora tenha sido observada uma redução nos níveis de proteína após o processamento, os valores permaneceram acima do limite aceitável. Todas as amostras foram avaliadas qualitativamente e resultaram em reprovação na segunda etapa do estudo, uma vez que os níveis se mantiveram superiores a 1 µg/cm^2^. Além disso, a presença de sujidade visível em parte das amostras após o processamento indica que a limpeza não foi totalmente eficaz na remoção de resíduos orgânicos.

## Data Availability

Os dados da pesquisa estão disponíveis no corpo do artigo.
